# The effect of contrast agents on left ventricular parameters calculated by a threshold-based software module: does it truly matter?

**DOI:** 10.1007/s10554-019-01587-9

**Published:** 2019-04-29

**Authors:** Andrea Szűcs, Anna Réka Kiss, Ferenc Imre Suhai, Attila Tóth, Zsófia Gregor, Márton Horváth, Csilla Czimbalmos, Ibolya Csécs, Zsófia Dohy, Liliána Erzsébet Szabó, Béla Merkely, Hajnalka Vágó

**Affiliations:** Heart and Vascular Center, Semmelweis University of Budapest, Budapest, Hungary

**Keywords:** Cardiac magnetic resonance, Left ventricular noncompaction, Contrast agent, Threshold-based trabecular quantification

## Abstract

The acquisition of short-axis (SA) cine magnetic resonance (MR) images after the administration of contrast agent (CA) is a common, time-saving technique, but a decreased difference in the blood-myocardium contrast on these steady-state free precession (SSFP) cine scans could change the calculated parameters when using threshold-based papillary and trabecular muscle (PTM) quantification. We studied the effect of CA on the parameters calculated from pre- and post-CA SA cine images in noncompaction cardiomyopathy (NC-CMP) and healthy (H) participants using a threshold-based module. A total of 39 individuals (20 patients and 19 healthy) were included prospectively in this study. After the pre-CA SA images were acquired, i.v. gadobutrol (GA) or gadobenate dimeglumine (GD) (GA vs. GD: NC-CMP = 12 vs. 8; C = 12 vs. 7) was administered, and SA scans were repeated after two minutes. A threshold-based PTM software was used for postprocessing. Pre-CA and post-CA SA images were analyzed, and the parameters were compared in both the NC-CMP and H groups. The left ventricular volumes were significantly larger, while the left ventricular myocardial (LVmass) and trabecular mass (LVtrab) values were significantly smaller on the post-CA scans (NC-CMP: pre-CA vs. post-CA, EDV: 74.0 ± 13.6 vs. 81.1 ± 16.3 ml/m^2^, ESV: 25.3 ± 7.3 vs. 30.1 ± 11.2 ml/m^2^, LVmass-ED: 82.5 ± 17.5 vs. 75.7 ± 15.9 g/m^2^, LVtrab-ED: 25.0 ± 6.6 vs. 18.9 ± 4.7 g/m^2^; Healthy: preCA vs. post-CA, EDV: 69.7 ± 11.9 vs. 72.2  ±  10.7 ml/m^2^, ESV: 22.6 ± 5.7 vs. 23.9 ± 6.3 ml/m^2^, LVmass-ED: 71.3 ± 13.6 vs. 68.7 ± 13.9 g/m^2^, LVtrab-ED: 19.4 ± 2.6 vs. 16.2 ± 3.0 g/m^2^; p < 0.05). The decreased blood-myocardium contrast difference on post-CA SSFP SA cine images leads to altered cardiac parameters when using threshold-based software for evaluation.

## Introduction

Postprocessing software has been developing continuously since cardiac magnetic resonance (CMR) imaging was first performed; thus, the currently available techniques are more user-friendly and are able to provide gold-standard data on cardiac function and mass [1]. One of the newest achievements of this technical development is threshold-based papillary and trabeculated muscle (PTM) quantification. Based on the different signal intensities of blood and myocardial tissue, these algorithms differentiate small endocardial trabeculae and papillary muscles from the blood pool without endocardial contours, resulting in more accurate data concerning volumetric parameters and myocardial mass [2, 3]. These algorithms can play a role in postprocessing in patients with myocardial disorders with hypertrabeculation, as inclusion or exclusion of excessive endocardial trabeculae during traditional contouring can significantly change the measured volumes and mass [4, 5]. In addition to the accuracy of the calculated data, the benefits of this approach include the ability to use the technique retrospectively without extra CMR images; the reproducibility of this approach is also excellent and independent of the experience of the observers [6].

Noncompaction cardiomyopathy (NC-CMP) is a rare disorder caused by the failure of myocardial compaction during embryogenesis and results in excessive trabeculation, mainly in the apical part of the left ventricle. The clinical significance of this disease is controversial; in many cases, left ventricle noncompaction ends in dilated cardiomyopathy and heart failure, but patients can also remain symptomless with good left ventricular ejection fraction values [7].

**Fig. 1:**
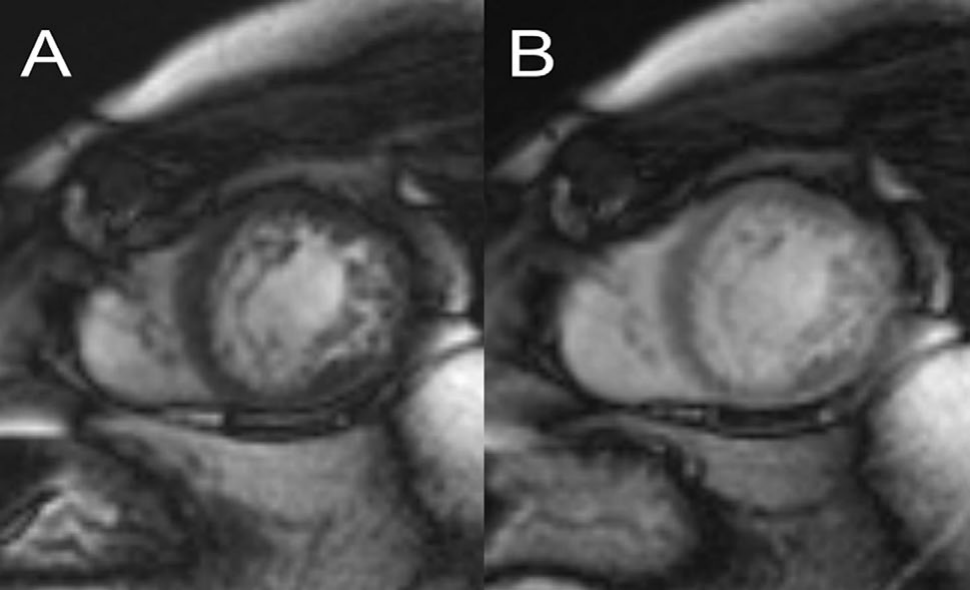
Short axis images collected before (**a**) and after (**b**) injection of contrast agent

NC-CMP has been diagnosed at a higher rate in recent years due to the wider availability of CMR. To shorten the scan time, SSFP short-axis (SA) cine images are often acquired after contrast agent (CA) administration, which is a global practice and a recommended technique for CMR examinations [8–10]. Endocardial trabeculation is harder to visualize on these scans because the CA alters the signal intensity of the blood pool and the myocardium on SSFP images, decreasing the difference between them. Therefore, the calculated volumetric data and the myocardial mass can be altered (Fig. 1). Moreover, the significance of this effect on the precision of another type of CMR analytical software, namely, feature tracking, has been reported in the literature [11].

Because limited data are available regarding the effect of CAs on postprocessing evaluation, we studied quantitative differences. The aim of our study was to quantify the effect of CA on calculated parameters using postprocessing software capable of threshold-based PTM quantification. First, pre- and postcontrast SSFP SA cine images of patients with left ventricular hypertrabeculation that were analyzed with this software were compared. Furthermore, we tested whether the loss of contrast difference between the blood and the myocardium caused alterations in the calculated parameters of a healthy normal population or whether this change plays a role only in patients with hypertrabeculation. Third, different types of gadolinium-based CAs show different relaxivity effects and slightly different kinetics; thus, we studied the effects of different contrast agents.

## Materials and methods

### Patient characteristics

Twenty NC-CMP patients with good left ventricular ejection fraction values and without any additional cardiac abnormalities or cardiovascular diseases and 19 healthy individuals were prospectively enrolled in 2016–2017. The exclusion criteria were the presence of congenital heart disease, ischemic heart disease, other cardiomyopathies or myocarditis in the patient’s history. The baseline parameters of NC-CMP patients and healthy individuals are reported in Table 1. All procedures performed in this study were in accordance with the ethical standards of the institutional and national research committee and with the 1964 Helsinki declaration and its later amendments or comparable ethical standards. Ethical approval was obtained from the Central Ethics Committee of Hungary, and all participants provided informed consent.

**Table 1:** Baseline characteristics of the noncompaction cardiomyopathy and healthy study groups

	NC-CMP n = 20	Healthy n = 19	p
Gadobutrol (n)	12	12	
Gadobenate dimenglumine (n)	8	7	
Age (years)	41.7 ± 16.3	37.9 ± 16.6	0.4736
EF (%)	66.1 ± 5.2	67.8 ± 5.5	0.9747
EDV (ml/m^2^)	74.0 ± 13.6	69.7 ± 11.9	0.3013
ESV (ml/m^2^)	25.3 ± 7.3	22.6 ± 5.7	0.1957
LV mass-ED (g/m^2^)	82.5 ± 17.5*	71.3 ± 13.6*	0.0316*
LV trab-ED (g/m^2^)	25.0 ± 6.6*	19.4 ± 2.6*	0.0016*

The parameters are converted to body surface area

*EDV* end-diastolic volume, *EF* ejection fraction, *ESV* end-systolic volume, *LV mass*-*ED* left ventricular end-diastolic myocardial mass, *LV trab*-*ED* left ventricular end-diastolic papillary and trabecular mass, *NC*-*CMP* noncompaction cardiomyopathy

*p < 0.05

### Image acquisition and study protocol

**Fig. 2:**
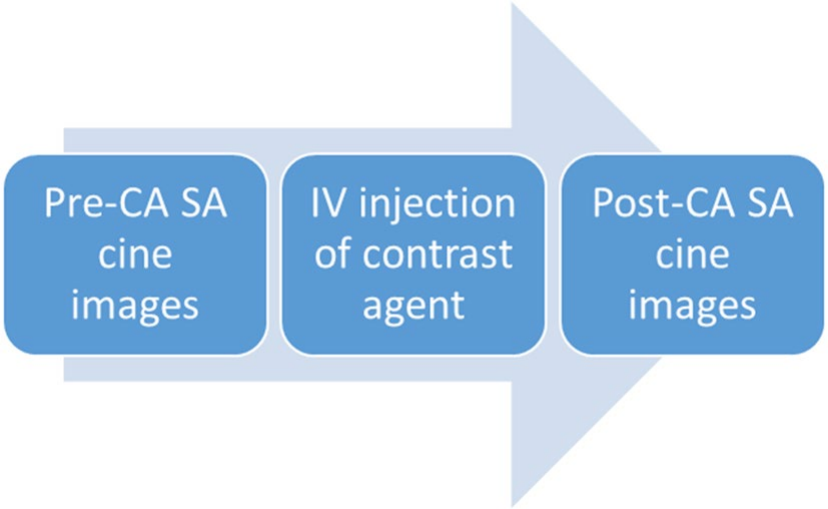
Study protocol. Pre-CA: pre-contrast agent, post-CA: post-contrast agent, SA: short axis

CMR imaging was performed on the participants within 1 year using a 1.5 T MR scanner (Achieva, Philips Medical Systems, Eindhoven, The Netherlands) and a 5-channel cardiac coil. Retrospectively gated, balanced steady-state free precession (bSSFP) cine images were acquired in conventional 2-, 3-, and 4-chamber long-axis views. Breath-hold SA cine images from base to apex were obtained. The slice thickness was 8 mm with no gap. After the SA cine images were acquired, either gadobutrol ((GA), Gadovist, Bayer-Schering, 0.16 ml/kg) or gadobenate dimenglumine ((GD), MultiHance, Bracco, 0.25 ml/kg) was injected intravenously. Each included individual received only one type of CA, which was decided randomly. GA was administered to 12 NC-CMP patients and 12 healthy normal participants, and GD was administered to 8 NC-CMP patients and 7 healthy participants (Table 1). After the contrast material was injected, another set of SA cine images was started after 2 min in the same location (Fig. 2).

### Image analysis

Semiautomatic tracing with manual correction of the epicardial borders was performed on SA images and was corrected by one medical professional (A. Sz., 7 years of experience) with excellent intraobserver agreement. The global concordance correlation coefficient, which represents the intraobserver agreement of all measured left ventricular parameters, was 0.88 (interpreted as: greater than 0.75 excellent).

**Fig. 3:**
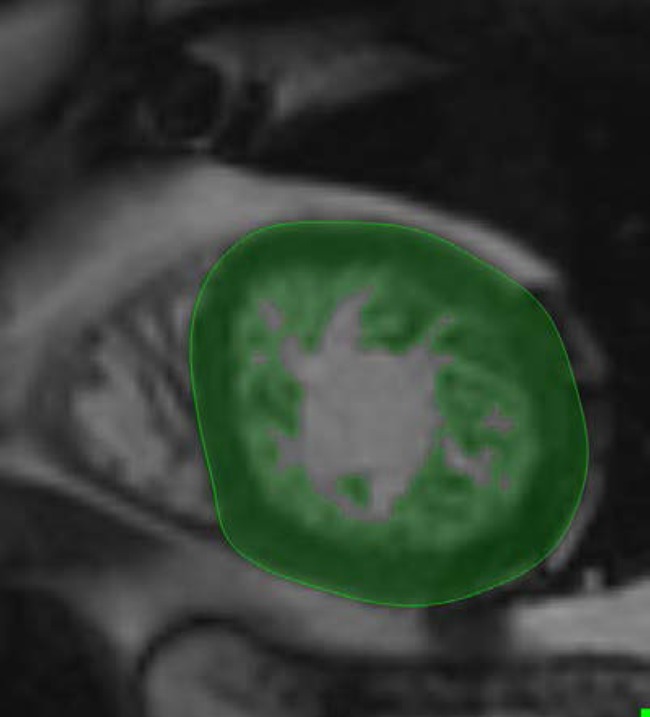
Image analysis with the threshold-based papillary and trabeculated muscle quantification software. The green area represents the myocardial mass including the endocardial trabeculation of the left ventricle

We used threshold-based PTM quantification analytical software (the MassK module of 7.6 QMass Medis, Leiden, The Netherlands) for quantitative image analysis. This algorithm calculates the blood percentage value of each pixel based on the differing signal intensities of the blood pool and myocardial tissue [2, 3]. The signal intensity threshold was set to the default (50%) (Fig. 3). Both the first (before CA administration (pre-CA)) and the second (after CA administration (post-CA)) SA scans were analyzed.

The following left ventricular parameters were calculated and converted to body surface area: end-systolic volume (ESV), end-diastolic volume (EDV), ejection fraction (EF), left ventricular end-diastolic myocardial mass (LVmass-ED), and left ventricular end-diastolic papillary and trabecular mass (LVtrab-ED). The normal left ventricular dimensions provided by Alfakih et al. were used as reference data which were established without the administration of contrast agent [12].

### Statistical analysis

The Shapiro–Wilk test was applied to assess the normality of the distribution of the data. All data are described as the mean and standard deviation. A paired-sample t-test was used to assess differences in parameters that had a normal distribution; otherwise, the Wilcoxon rank sum test was used. p values less than 0.05 were considered significant. MedCalc Statistical Software version 17.9.5 (MedCalc Software, Ostend, Belgium) was used for statistical calculations.

## Results

### Comparison of the pre- and post-CA scans

We compared the parameters calculated from the pre-CA and post-CA scans both in the NC-CMP and healthy groups and found significant differences in the left ventricular parameters; the EDV and ESV were significantly larger, and the left ventricular myocardial mass and trabecular mass were significantly smaller on the post-CA scans in both groups (Table 2). Next, the values of the post-CA parameters were subtracted from the values of the pre-CA parameters, and the absolute values were used to compare the differences between the pre- and post-CA parameters of the two groups; the difference between the scans was significantly larger in the NC-CMP group than in the healthy normal group (NC-CMP difference vs. healthy normal difference: EDV, 7.9 ± 6.0 vs. 3.3 ± 3.3 ml/m^2^; ESV, 5.4 ± 6.3 vs. 2.0  ± 2.1 ml/m^2^; EF, 3.3 ± 2.7 vs. 2.3 ± 2.7%; LVmass-ED, 7.0 ± 4.8 vs. 3.3 ± 2.1 g/m^2^; LVtrab-ED, 6.0 ± 3.9 vs. 3.2 ± 1.7 g/m^2^; p < 0.005).

**Table 2:** Calculated parameters of the noncompaction cardiomyopathy and healthy study groups calculated from pre-contrast and post-contrast scans

NC-CMP				Healthy		
	Pre-CA	Post-CA	p	Pre-CA	Post-CA	p
ESV (ml/m^2^)	25.3 ± 7.3	30.1 ± 11.2	0.0059*	22.6 ± 5.7	23.9 ± 6.3	0.0411*
EDV (ml/m^2^)	74.0 ± 13.6	81.1 ± 16.3	0.0002*	69.7 ± 11.9	72.2 ± 10.7	0.0121*
EF (%)	66.1 ± 5.2	64.7 ± 5.8	0.1608	67.8 ± 5.5	67.1 ± 6.4	0.7086
LV mass-ED (g/m^2^)	82.5 ± 17.5	75.7 ± 15.9	< 0.0001*	71.3 ± 13.6	68.7 ± 13.9	0.0010*
LV trab-ED (g/m^2^)	25.0 ± 6.6	18.9 ± 4.7	< 0.0001*	19.4 ± 2.6	16.2 ± 3.0	< 0.0001*

The parameters are converted to body surface area

*EDV* end-diastolic volume, *EF* ejection fraction, *ESV* end-systolic volume, *LV mass*-*ED* left ventricular end-diastolic myocardial mass, *LV trab*-*ED* left ventricular end-diastolic papillary and trabecular mass, *NC*-*CMP* noncompaction cardiomyopathy, *post*-*CA* post- contrast agent, *pre*-*CA* pre- contrast agent

*p < 0.05

### Comparing the effects of different CAs

Since different types of CAs are in use, we tested whether these agents have similar effects on the studied parameters. We first compared the pre- and post-CA results in the NC-CMP group (pre-GA vs. post-GA and pre-GD vs. post-GD). We found results similar to those obtained in the comparison of the total pre-CA versus post-CA scans. Regardless of the applied contrast material, the EDV, ESV and EF values were significantly larger, while the LVmass-ED and LVtrab-ED values were significantly smaller when calculated from the post-CA scans (Table 3). We performed these comparisons in the healthy normal group as well and obtained similar results; the LVtrab-ED values were significantly lower in the post-CA scans for both contrast materials. LVmass-ED values were also significantly lower in the post-GA scans, and EDV values were significantly higher in the post-GD scans (Table 3).

**Table 3:** Comparing the effect of gadobutrol (GA) and gadobenate dimenglumine (GD) on the calculated parameters

	Pre-GA	Post-GA	p	Pre-GD	Post-GD	p
NC-CMP						
ESV (ml/m^2^)	24.1 ± 7.2	28.9 ± 12.6	0.0269*	27.2 ± 7.6	31.8 ± 9.0	0.0013*
EDV (ml/m^2^)	71.7 ± 12.7	79.5 ± 17.8	0.0076*	77.4 ± 15.0	83.6 ± 14.6	0.0046*
EF (%)	66.6 ± 5.8	66.3 ± 5.3	0.8025	65.3 ± 4.4	62.4 ± 6.0	0.0425*
LV mass-ED (g/m^2^)	81.4 ± 19.6	75.6 ± 17.4	0.0031*	84.3 ± 15.0	75.9 ± 14.5	0.0008*
LV trab-ED (g/m^2^)	24.7 ± 7.8	18.8 ± 5.1	0.0014*	25.4 ± 4.9	19.1 ± 4.4	0.0001*
Healthy						
ESV (ml/m^2^)	22.5 ± 5.0	23.9 ± 6.1	0.0771	22.7 ± 7.1	23.9 ± 7.1	0.3054
EDV (ml/m^2^)	69.2 ± 9.5	71.2 ± 7.2	0.1627	70.5 ± 16.2	73.9 ± 15.6	0.0075*
EF (%)	67.5 ± 5.9	66.7 ± 6.9	0.7334	68.2 ± 5.1	67.9 ± 5.7	0.8034
LV mass-ED (g/m^2^)	71.3 ± 12.3	68.4 ± 12.8	0.0065*	71.3 ± 16.6	69.1 ± 16.5	0.0993
LV trab-ED (g/m^2^)	19.7 ± 2.2	16.4 ± 3.0	0.0001*	19.0 ± 3.4	15.9 ± 3.4	0.0015*

The parameters are converted to body surface area

*EDV* end-diastolic volume, *EF* ejection fraction, *ESV* end-systolic volume, *LV mass*-*ED* left ventricular end-diastolic myocardial mass, *LVtrab*-*ED* left ventricular end-diastolic papillary and trabecular mass, *NC*-*CMP* non-compaction cardiomyopathy, *post*-*GA* post-gadobutrol, *post*-*GD* post- gadobenate gimenglumine, *pre*-*GA* pre-gadobutrol, *pre*-*GD* pre-gadobenate dimenglumine

*p < 0.05

### Comparison of the CAs to each other

Finally, we compared the GA and GD CAs to each other in the healthy and NC-CMP groups (pre-GA vs. pre-GD). Because the parameters calculated from the pre-CA scans did not differ significantly between the healthy and patient groups, we compared the post-CA scans and found no significant difference between the groups (Table 4).

**Table 4:** Comparison of the gadobutrol (GA) and gedobenate dimenglumine (GD) receiving populations’ pre-and post-contrast scans

	Pre-CA			Post-CA		
	GA	GD	p	GA	GD	p
NC-CMP						
ESV (ml/m^2^)	42.3 ± 10.2	46.7 ± 8.1	0.9453	44.1 ± 10.8	49.6 ± 9.1	0.8438
EDV (ml/m^2^)	73.4 ± 11.3	77.4 ± 15.0	0.5701	81.5 ± 18.9	83.6 ± 14.6	0.8299
EF (%)	65.4 ± 6.3	65.3 ± 4.4	0.9484	64.4 ± 5.1	62.4 ± 6.0	0.5706
LV mass-ED (g/m^2^)	82.8 ± 17.2	84.3 ± 15.0	0.8513	77.0 ± 14.0	75.9 ± 14.5	0.8899
LV trab-ED (g/m^2^)	24.0 ± 6.3	25.4 ± 4.9	0.8919	19.4 ± 4.6	19.1 ± 4.4	0.8919
Healthy						
ESV (ml/m^2^)	24.6 ± 3.8	22.7 ± 7.1	0.5245	25.5 ± 4.5	23.9 ± 7.1	0.6847
EDV (ml/m^2^)	72.1 ± 9.1	70.5 ± 16.2	0.8346	72.8 ± 8.1	73.9 ± 15.6	0.8715
EF (%)	65.6 ± 5.1	68.2 ± 5.1	0.4415	65.0 ± 4.5	67.9 ± 5.7	0.4459
LV mass-ED (g/m^2^)	72.5 ± 12.1	71.3 ± 16.6	0.8756	72.8 ± 8.1	73.9 ± 15.6	0.8715
LV trab-ED (g/m^2^)	19.7 ± 2.2	19.0 ± 3.4	0.6321	16.0 ± 2.6	15.9 ± 3.4	0.9451

The parameters are converted to body surface area

*EDV* end-diastolic volume, *EF* ejection fraction, *ESV* end-systolic volume, *LV mass*-*ED* left ventricular end-diastolic myocardial mass, *LVtrab*-*ED* left ventricular end-diastolic papillary and trabecular mass, *NC*-*CMP* non-compaction cardiomyopathy, *post*-*GA* post-gadobutrol, *post*-*GD* post- gadobenate gimenglumine, *pre*-*GA* pre-gadobutrol, *pre*-*GD* pre-gadobenate dimenglumine

*p < 0.05

## Discussion

CMR imaging is currently the gold standard for measuring cardiac volume and function and myocardial mass [13–16]. In recent years, an increasing number of postprocessing programs have been equipped with threshold-based papillary and trabeculated muscle-quantifying algorithms, leading to easier and faster evaluation and more accurate cardiac volumes and masses [2, 3].

This study was designed to confirm our experience with the postprocessing evaluation of scans made after the injection of gadolinium-based contrast material, namely, that endocardial trabeculation is harder to detect. We studied the effect of CAs on the applicability of threshold-based PTM quantification software in patients with left ventricular noncompaction and in healthy normal study subjects.

Our results showed that the EDV and ESV values calculated from post-CA scans were significantly higher, while the ED-mass and LVtrab-ED values calculated from post-CA scans were significantly lower than those calculated from pre-CA images in the NC-CMP and healthy normal groups. However, the difference between the pre-CA and post-CA parameters was significantly larger in the patient group than in the healthy group.

The signal intensity of SSFP images depends on the T2/T1 ratio of the tissue of interest. Gadolinium-based extracellular CAs decrease the T1 values of blood and myocardial tissue, which results in increased signal intensity on SSFP images. This effect is more pronounced in the myocardium and less pronounced in the blood pool, leading to decreased contrast between the two tissues. T2 values are slightly reduced by CAs administered at low doses (0.1–0.3 mmol/kg), and these changes are overridden by T1 shortening; thus, changes in signal intensity after administration of a CA are due to changes in T1 values [17–19]. The end result of these changes in relaxivity is that the difference between the T1/T2 ratios of the blood and the myocardium decreases after contrast administration. As the mechanism of threshold-based quantification is based on the high signal intensity difference between the blood and the myocardium, our results suggest that this effect has a significant impact on the detection of endocardial trabeculae on postcontrast scans, not only in patients with left ventricular hypertrabeculation but also in patients with normal trabeculation. Our results correlate with those from a study about the precision of another technique (feature tracking) from a different vendor on post-CA scans; in that study, a contrast agent significantly changed the measured strain values, which also confirmed the importance of the signal intensity-altering effect of contrast agents [11]. These results are important both during regular CMR postprocessing and in research projects for standardizing protocols.

We also studied the effect of two different contrast materials on the precision of threshold-based software to determine whether the change in the calculated parameters depends on the type of CA applied. The LVtrab-ED value was significantly smaller and the EDV value was larger on both post-GA and post-GD images. However, no significant differences were found in the comparison between the two CAs.

Gadolinium is a paramagnetic extracellular CA that shortens the T1 and T2 relaxation time of the surrounding protons, which increases signal intensity on T1-weighted images [20]. Different chelators are used to create complexes with gadolinium; therefore, different products are available. Compared to traditional extracellular gadolinium CAs, GD binds weakly to albumin and in this way attenuates the signal intensity of blood, has a slight intravascular effect and prolongs the plasma half-life. GA does not bind to proteins but reduces T1 values more than GD because of its concentration [21–23]. These properties do not seem to make a significant difference regarding the effect studied here.

In summary, this study reveals that the threshold-based PTM-quantifying software provides altered results when SA scans are made after the injection of contrast material, as the signal intensity difference between the blood and the myocardium is decreased on these postcontrast images. This effect is independent of the type of CA applied and the amount of endocardial trabeculation. In this study, CAs influenced the measured values, especially in patients with excessive endocardial trabeculation; therefore, the method of evaluation should be standardized. Further studies are required to evaluate this phenomenon with myocardial hypertrabeculation.

## Limitations

The main limitation is the small number of included patients, as noncompaction cardiomyopathy is a relatively rare disease. Furthermore, some patients did not consent to receive the CA; thus, we could not include those patients.
